# *Bombyx mori* cocoon as a promising pharmacological agent: A review of ethnopharmacology, chemistry, and biological activities

**DOI:** 10.1016/j.heliyon.2022.e10496

**Published:** 2022-09-01

**Authors:** Hossein Biganeh, Mahdi Kabiri, Yahya Zeynalpourfattahi, Rose Meire Costa Brancalhão, Mehrdad Karimi, Mohammad Reza Shams Ardekani, Roja Rahimi

**Affiliations:** a Department of Pharmacognosy, Faculty of Pharmacy, Baqiyatallah University of Medical Sciences, Tehran, Iran; b PhytoPharmacology Interest Group (PPIG), Universal Scientific Education and Research Network (USERN), Tehran, Iran; c Department of Traditional Pharmacy, School of Persian Medicine, Tehran University of Medical Sciences, Tehran, Iran; d Department of Traditional Medicine, School of Persian Medicine, Tehran University of Medical Sciences, Tehran, Iran; e Center of Biological Sciences and Health, State University of Western Paraná, Rua Universitária, 2069, 85819-110, Cascavel, PR, Brazil; f Department of Pharmacognosy, Faculty of Pharmacy, Tehran University of Medical Sciences, Tehran, Iran

**Keywords:** Silk cocoon, Aminoacid, Protein, Fibroin, Sericin, Heart

## Abstract

Silk cocoon, naturally produced by silkworms scientifically named *Bombyx mori* L. (Lepidoptera, Bombycidae), is one of the well-known medicinal agents with several therapeutic activities. The present study aims to review the various aspects of the silk cocoon, including chemical composition, traditional uses, biological and biotechnological activities, and toxicological issues, to provide a scientific source for scholars. For this purpose, Electronic databases including PubMed, Scopus, Google Scholar, Web of Science, and traditional literature, were searched up to December 2021. According to the historical data, silk farming is acknowledged as one of the most ancient agricultural findings. The silk is generally composed of 75–83% fibroin, 17–25% sericin, and 1–5% non-sericin components, including secondary metabolites, wax, pigments, carbohydrates, and other impurities. Flavonoids, especially quercetin and kaempferol, alkaloids, coumarin derivatives, and phenolic acids, are among the secondary metabolites isolated from the silk cocoon. In recent years the biological properties of the silk cocoon, especially its major proteins, namely fibroin and sericin, have drawn special attention. Scientific literature has investigated several pharmacological effects of the silk cocoon and its ingredients, including cardioprotective, antioxidant, anticancer, antidiabetic, antihyperlipidemia, gastroprotective, as well as ameliorated skin health activities. In addition, it has been extensively taken into consideration in drug delivery and tissue engineering study fields. Furthermore, its toxicity is in acceptable range.

## 1 Introduction

Silk is a natural fiber produced by arthropods like spiders, silkworms, and scorpions; among them, the silk derived from the domesticated silkworm, *Bombyx mori* L*.* (Lepidoptera, Bombycidae), is widely exploited [1]. Regarding historical data gleaned from North China, since 5000 years dating back, commercial silk farming has been put forth as one of humanity's most ancient agricultural findings [2]. Besides, silk has been abundantly used traditionally in diverse cultures as a natural therapy or yarn-related trade [3].

The silk cocoon layer is constructed from two major proteins: fibroin and sericin, in which fibroin, the fibrous protein, is the central part, and sericin, the globular protein, is the sticky part that envelopes the fibers and coheres them together. Furthermore, impurities like carbohydrates, salts, and waxes known as "non-sericin" constituents impart water repellency to the silk cocoon [4]. It is estimated that out of approximately 1 million tons of globally produced fresh cocoons, roughly 400000 tons of dry cocoons are achieved that yielding 50000 tons of sericin [5]. Sericin has been neglected for years; so that to prepare smooth, soft, and lustrous silk filaments from silkworms' cocoons for the textile industry, sericin is moved apart from fibroin in the so-called "degumming" or "refining" process using different chemical, enzymatic, or hydrothermal methods, and it is dominantly discarded in wastewater [4, 6]. Apart from its environmental concerns posed by different pollution discarded in wastewater, sericin removal poses some challenges like a high volume of oxygen needed for its degradation by microorganisms and burden of economic issues, especially in countries where silk farming is extensively taking place like India and China [2, 4]. Furthermore, because of its diversity of amino acids and functional groups, biocompatibility, and biodegradability, sericin has recently drawn special attention among scholars to study more about its biomedical capabilities [3]. In this sense, sericin has exhibited a broad range of health-promoting activities, namely, antimicrobial, antioxidant, anticancer, coagulant, antityrosine, UV protection, as well as humidifying activities; in which the latter has endorsed it as an appropriate option in skin health and toiletries [7]. FDA has formerly introduced sericin and its derivatives as a “Generally recognized as safe” substance [8]. Likewise, silk, particularly fibroin silk, has been used in medical commodities, food additives, novel drug delivery methods, and scaffold development for tissue engineering of different organs due to their biocompatibility and a broad spectrum of outstanding physiochemical features [9, 10].

Since there is no comparative review study on therapeutic effects between traditional and new medical use of this ancient drug, we deliberate on summing up the various prospects of the aqueous extract silk cocoon, including phytochemical constituents, pharmacological activities, traditional applications, and toxicological aspects, to provide a scientific source to plate future natural drugs elicited from this agent for managing various disorders.

## 2 Materials and methods

Databases, including PubMed, Scopus, Google Scholar, Web of Science, and traditional literature, were searched. The included search words were “silk AND/OR sericin AND/OR fibroin, AND/OR abrisham AND/OR abresham” and the articles represent phytochemistry OR pharmacological activity OR traditional uses OR toxicity of silk cocoons are included in this review. Data were collected from 1966 to 2021 (up to December). There was no language restriction. The reference list from retrieved papers was also reviewed for additional applicable studies. All published articles, as well as abstracts presented at meetings, were evaluated.

## 3 Chemical constituents of the silk cocoon

Regarding different types of silkworms in light of nutrition sources and components extraction methods, the cocoon is mainly composed of fibroin, sericin, and other impurities (e.g. pigments, waxes, carbohydrates, and phytochemicals), accounting for 75–83, 17–25, and about 1–4% of cocoon constituents, respectively [10]. The amino acid residues found in silk proteins could be functionally summed up into three classes comprising charged (aspartic acid), polar (serine), and hydrophobic (glycine) amino acids [11].

Silk fibroin secreted from the posterior section of the labile gland of silkworm is made of three proteins namely H-chain, low-chain, and glycoprotein P25 with 350, 26, and 30 kDa molecular weight, respectively [12]. It is constructed from 18 out of 20 known amino acids which non-polar amino acids, such as glycine, alanine, and valine, account for 76% of the protein, while others, comprising polar amino acids, especially serine, accounted just for roughly 24% [4].

Silk sericin is a greatly hydrophilic macromolecular family of glycoproteins produced by the middle silk gland that applies 25–30% of the cocoon weight [2]. Sericins’ molecular mass spans from 24 to 400kDa, and like fibroin, it is made of 18 amino acids possess a prominent number of polar functional groups like hydroxyl, carboxyl, and amino groups, permitting the formation of crosslinks, copolymerization, and joining with different polymers [13]. In spite of the amino acid composition of fibroin, silk sericin is majorly composed of polar amino acids such as serine and aspartic acid. Moreover, sericin has existed in various secondary configurations, namely β-sheet or random coil, which changes between them are readily achievable in response to factors such as tensile forces, hydrolytic degradation, and/or temperature [2]. However, despite research studies in the quest for the composition and structure of the proteins, there is no unified consensus until now [14].

**Figure 1:**
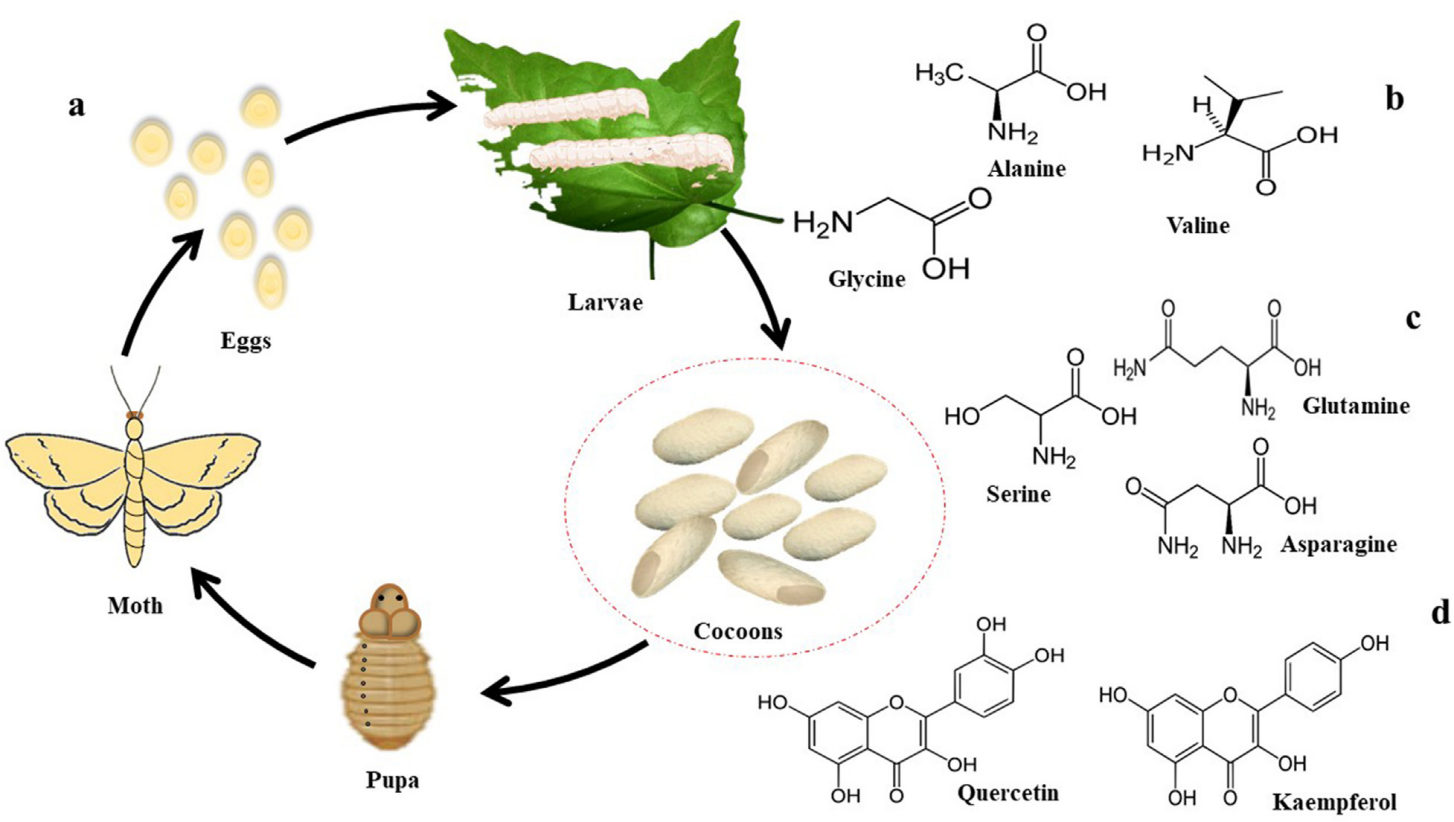
Major chemical constituents of silkworm cocoons. a: silkworm lifecycle. b: major amino acids found in silk fibroin and c: silk sericin, d: secondary metabolites found in silk cocoons.

Phytochemicals are naturally occurring secondary metabolites with a broad spectrum of varieties and health-promoting properties ubiquitous in nature [15]. In this scene, different types of flavonoids, especially quercetin and in a lesser amount kaempferol both in free or glycosylated forms [16], alkaloids [17], and coumarin derivatives [18], along with aromatic ethers, namely 3,4-dihydroxyphenyl-n-pentanyl ether and 2,3,4-trihydoxypenyl-n-pentanyl ether [19] have been isolated from the silk cocoons. Besides, for the first time, two naturally occurring flavonoids containing amino acid moiety have been isolated from the cocoon shell of *B. mori* L. silkworm [20]. Figure 1 demonstrates some major chemical constituents of silk cocoons.

## 4 Silk in traditional medicine

Concerning historical data, silk farming is regarded as an ancient agricultural discovery found by humans. This was in attendance from 5000 years ago in North China, where it was distributed on other sides of the world [2]. However, Archaeological evidence reveals that silk dates back to more than a millennium earlier in South Asia, especially the Indus region, than China when it is deemed that *Bombyx mandarina*, the wild silk moth, was domesticated in China into the well-known *B. Mori* [21].

Silks are well-known polymers used as sutures since ages [3]. In Persian medicine literature, the silk cocoon is called abrisham (abresham), which is generally known as abresham muqriz (muqriz means cut). Abrisham is one of the 60 natural remedies that Avicenna referred to in his treatise on cardiovascular disease [22].

Abrisham with different ingredients from natural sources formulated in various reputed poly pharmaceutical Unani formulations; namely, Khameer-e- Abresham Sada, Khameere Abresham Hakeem Arshad Wala, etc., are used to treat various cardiac and nervous ailments [19, 22]. Regarding ethnomedicinal uses in South India, silkworm ash and cocoon have been used as a rejuvenating tonic and aphrodisiac [23].

In traditional Persian medicine, silk is widely used in the treatment of respiratory and cardiovascular disorders [22]. Mulberry silkworm cocoons carbonisata as a charcoal-based drug in traditional Chinese medicine used to relieve pain and halt bleeding, were first described in the Peaceful Holy Benevolent Prescriptions, the official medical book of the early Chinese Song Dynasty imperial (960–1127 A.D.). For hundreds of years, due to its outstanding bioactivity and safety profile, the carbonized Mulberry silkworm cocoons have been broadly exploited in alleviating disorders such as skin ulcers, fever, fatigue, etc. [24]. Furthermore, silk cocoons' potential in biological systems health such as reducing blood pressure and heart palpitation, bronchodilation and asthma alleviating, as well as treating eye catarrh has been noticed [19].

## 5 Pharmacological effects

Many studies have been accomplished regarding the beneficial therapeutic properties of silkworm cocoon whole extract and its isolated components, some of which agree with the effects noted in the traditional Iranian literature. Both in vitro and in vivo activities are discussed herein and also in details in Table 1.

**Table 1:** Pharmacological activities of silk cocoon.

Pharmacological activity	Intervention	Dose	Duration (weeks)	Model	Results	Reference
Cardiovascular activity	Different traditional formulations from cocoon silk extract	-	∼4 (30 days)	Isoproterenol-induced cardiotoxicity in rats	Suppression of heart damage through ↓cardiac marker enzymes (CK-MB and troponin), heart weight to body weight ratio, degree of myonecrosis and filtration of inflammatory cells, and lipid peroxidation; Improvement of heart recovery and cardiac antioxidant capacity	[25]
	Pretreatment by ethanolic extracts of silk cocoon	250 and 500 mg/kg/d	4	Isoprenaline-induced myocardial infarction in rats	↓inflammation, redness, capillary dilation, and scar formation in histopathologic findings; Improvement of various cardiac enzymes and heart weight/body weight ratio	[26]
	Pretreatment with traditional formulation (Khamira Abresham Hakim Arshad Wala)	200 mg/kg/d	1	Doxorubicin-induced cardiotoxicity in rats	↓cardiac-related enzymatic changes and tissue damages; ↑antioxidant activities (reinstatement of MDA and GSH levels)	[27]
Anti-hyperlipidemic activity	Silk protein with different fibroin/sericin compositions: F100 (pure fibroin), F81 (81:19 fibroin/sericin, w/w) or F50 (50:50 fibroin/sericin, w/w)	-	6	High fat-fed mice	↓body weight and enhanced lipid profile; ↓body fat, TG and total plasma chol levels, atherogenic index and free fatty acid level; ↑HDL-C level; ↑fecal lipid excretion, inhibition of lipogenesis, and regulation of adipokine production; These effects were increased by increasing the amount of sericin in the diet	[28]
	Silk cocoon extract	500 mg/kg/d	6	Cholesterol diet-induced hyperlipidemia and atherosclerosis in rabbits	↓atherosclerotic plaques size; ↑body weight and HDL-C levels	[29]
	Sericin-derived oligopeptides	50–1000 μg/ml	-	Caco-2 cell line	↓chol uptake in the cell line	[30]
	Sericin-derived oligopeptides with high fat diet	10, 50, and 200 mg/kg/d	4	High cholesterol-fed rats	↓total serum and non-HDL chol	
	Silk sericin with high fat diet	25 and 50 μg/ml	-	Caco-2 cell line	↓30% of chol uptake in the cell line	[31]
		10, 100, and 1000 mg/kg/d	2	High cholesterol-fed rats	↓total serum and non-HDL chol	
	Sericin with high fat diet	4% w/w	5	High fat-fed rats	↓serum levels of TG, chol, phospholipids, and free fatty acids; ↓VLDL-TG, VLDL-C, LDL-C, and LDL-phospolipids; ↓liver TG and lipogenic enzymes like glucose-6-phosphate dehydrogenase and malic enzyme; ↑serum adiponectin; final body and white adipose tissue weight remained unchanged	[32]
	Sericin with high fat diet	1,000 mg/kg/d	4	High-fat diet-induced obese mice	Not entirely recovery of biochemical and biometric changes induced following obesity induction; No impact on mice bowel transit time; ↑lipid excretion; restored intestinal wall morphometry;	[33]
Antioxidant and Antitumor activities	Pretreatment with sericin protein	0.375, 0.75, and 1.5 g/kg/d	∼4 (30 days)	Alcohol-induced hepatic injury in mice	↓alcohol concentration in serum; ↑urine level of alcohol; Restoration of elevated antioxidant enzymes like MDA, GSH, and SOD to normal values; Restoration of hepatic mitochondria to normal form; Normal histology with mild congestion of central vein at the dose of 1.50 g/kg	[34]
	Silk sericin	-	-	Enzymatic assay	Vigorous scavenging activity against hydroxyl, superoxide, and DPPH radicals; Potent antioxidant action on the peroxidation of linoleic acid; Significant reducing power and ferrous-ion-chelating activity	[35]
	Silk sericin hydrolysates obtained by different proteases	-	-	Enzymatic assay	Significant reducing power and ferrous-ion-chelating activity of all of the silk sericin hydrolysates; Pronounced radical scavenging and lipid peroxidation activity of alcalase hydrolysates than other proteases	[36]
	Silk sericin	35, 50, 100, and 150 ng/ml	-	Hydrogen peroxide-induced oxidative stress in the skin fibroblast cell line (AH927)	↓MDA, LDH, and catalase cellular release in the pre-incubated cells with sericin before H2O2 treatment; ↑viable cells at 35 ng/ml compared to H2O2-treated cells	[37]
	Silk sericin with 1,2-diethylhydrazine for the initial 10 weeks	30 g/kg/d	∼21 (115 days)	1,2-dimethylhydrazine-challenged mice	↓incidence of colon carcinoma; ↓number of colon adenomas; ↓BrdU labeling index of colonic proliferating cells; ↓expression of colonic c-myc and c-fos proteins; ↓levels of colonic 8-hydroxydeoxyguanosine, 4-HNE, and inducible nitric oxide synthase protein	[38]
	Silk sericin	25-1,600 mg/ml	-	Human colorectal cancer SW480 cell line	↓cell viability; triggering cell apoptosis through ↑caspase-3 and ↓Bcl-2 expression; Induction of cell cycle arrest at the S phase	[39]
	Treatment with silk sericin	3%/d	4	1,2-dimethylhydrazine-treated rats	↓colon mucosal lipid peroxide; ↓number of aberrant crypt foci in all areas of the colon; ↑antioxidant activity	[40]
	Topical sericin immediately after UVB treatment	5 mg/d	1	UVB-induced skin lesion in hairless mice	↓formation of skin lesion (↓area and intensity of red color of the lesions); ↓epidermal thickness; ↓PCNA; ↓4-HNE and COX-2 protein expression	[41]
	Topical sericin immediately after 7,12-dimethylbenz anthracene (DMBA) treatment followed 1 week later by UVB irradiation twice weekly for 22 weeks	5 mg/d	22	UVB-induced tumor promotion in the DMBA-initiated mouse skin	↓tumor incidence and multiplicity	
Gastrointestinal protective activity	Co-administration of beef tallow and sericin	40 g/kg/d	3	High fat-fed rats	↑fecal IgA and mucin (indices of intestinal immune and barrier functions, respectively); ↓acetate and n-butyrate cecal organic acids; No change in microflora profile in cecal digesta and fecal secondary bile acids between high fat-diet with or without sericin	[43]
	Pretreatment with sericin protein	0.2, 0.4, 0.8 g/kg/d	∼4 (30 days)	Alcohol-induced gastric injury in mice	↓alcohol concentration in serum; ↑alcohol concentration in urine; Reversion of gastric damage indicators including MDA, GSH, GSH-PX, and SOD; Gastric mucosal mitochondria restoration	[44]
	Pretreatment with sericin protein	40 g/kg/d	1	Atropine-induced constipation in rats	Notable decreased in the fecal wet and dry weight in the control group; No change in fecal wet or dry weight in sericin fed rats; Higher water content in sericin group than the control group (regardless of atropine administration)	[45]
	Co-administration of sericin, white egg albumin and different microelements	30 g/kg/d	∼2 (12 days)	Male Sprague-Dawley rats	↑apparent absorption of Zn, Fe, Mg and Ca; no change in their urinary excretion; ↑their bioavailability; No change in final body weight, food intake amount and fecal dry weight	[46]
Hypoglycemia and its related complications activities	SF peptides	50 mg/ml	-	Insulin resistant HepG2 cells	↑glucose and lipid metabolism; ↑glucose consumption; ↓TG levels; ↓ROS, MDA, TNF-α and IL-6; ↑SOD and catalase activity; ↑total antioxidant capacity; ↑glucose consumption and glycogen accumulation, whereas ↓TG levels, ROS, and MDA in SF and metformin (0.01 mg/ml) combination treatment compared to metformin or SF alone	[49]
	Acid-hydrolyzed silk peptides	0.05, 0.1 and 0.5 g/kg/d	8	Non-obese insulin-insufficient partial pancreatectomized (Px) rat model of T2DM	↑food efficiency and body weight gain; partially protection against Px-induced bone mineral density and lean body mass decrement; ↑oral glucose and maltose and insulin tolerance; ↑insulin secretory capacity	[50]
	Acid-hydrolyzed silk peptides with high-fat diet	50 and 200 mg/kg/d	6	High-fat diet-induced obesity	Inhibition of body mass gain and the expression of adipogenic transcription factors in subcutaneous (SAT) and visceral adipose tissue (VAT); ↓blood glucose and adipocyte size increment; ↑oral glucose tolerance; ↓HbA1c; ↑GLUT4 and UCP3 expression; ↓ubiquitin proteasome and promoted myoblast determination protein 1 (MyoD)/myogenic factor 4 (myogenin) expression	[51]
	Acid-hydrolyzed silk peptides	25, 50, 100, 200, and 400 μM	-	3T3-L1 adipocytes	↓lipid accumulation; ↓expression of the adipogenic markers (C/EBPα and PPARγ)	
	Ethanolic extract from the green cocoon sericin layer	150, 250, and 350 mg/kg/d	7	T2DM mice	Improved oral glucose tolerance and insulin tolerance; ↓blood glucose level; ↑insulin and HbA1C levels; ↑islet area and the number of insulin-positive beta cells; ↓HOMA-IR and ↑ISI; ↓NFκB, IL-6, and TNF-α; ↑SOD and GSH-Px activities;	[52]
	A peptide fraction of sericin hydrolysate	0–400 μg/ml	-	α-glucosidase inhibition enzymatic assay	Similar α-glucosidase inhibitory activity as acarbose	[53]
	Silk sericin hydrolysate	0.8%/d (g%)	4	T2DM mice	↓fasting blood glucose, fasting plasma insulin, and glycosylated serum protein levels; Improved oral glucose tolerance and insulin tolerance; Ameliorated damaged β-cells and the liver tissue; ↑expression of insulin receptor, insulin receptor substrate, PI3K, phosphorylated-AKT, hepatic kinase, GLUT4, glycogen synthase, GSK3β, GLK, PFK1, PKM2, and AMPKα (associated with insulin metabolism and glycolysis); ↓expression of G6Pase, PCK, and ACC, (associated with gluconeogenesis and lipid metabolism in the liver); ↓expression of TNF-α, IL-6, P65, and IKKβ (associated with inflammation); ↑antioxidative activities	[54]
	Hydrolyzed SF	20% drinking water and a mixture of hydrolyzed SF	6	Pancreatic β-Cells in the C57BL/KsJ-Lepr^db/db^ mice (in vivo T2DM model)	↑pancreatic β-cell numbers; ↑blood insulin level; ↓blood glucose concentration; No change in body weight; ↑the number of PCNA and the ratio of BrdU positive cells; ↓number of apoptotic cells; ↑the expression of transcription factors involved in β-Cells regeneration; ↑the number of insulin-positive cells	[55]
	Silk sericin	2.4 g/kg/d	5	T2DM rats	↓blood glucose concentration; ↓the expression levels of MKK6, p-p38MAPK, NF-κB, IL-1β, IL-6, NLRP3 and caspase-1; ↓pathological changes-related to diabetes induction; No change in p38MAPK expression	[56]
	Silk sericin	2.4 g/kg/d	5	T2DM rats	↓serum growth hormone levels; ↓growth hormone expression; ↑serum testosterone and IGF-1 levels; upregulation of testicular growth hormone receptor and IGF-1 expression; improved spermatogenic activity by regulating the growth hormone/IGF-1 axis	[57]
	Silk sericin instillation into the eyes of the rats five times a day following corneal abrasion	10%	3 days	Corneal wound-induced Otsuka Long-Evans Tokushima Fatty rats (T2DM model)	↑wound healing progression and wound size reduction; Complete wound healing 48 h after abrasion	[58]
	Silk sericin	2.4 g/kg/d	5	Sciatic-related nerve cells injuries in T2DM rats	↓serum blood glucose level; promotion of neurofilament protein expression in the sciatic nerve and nerve growth factor in L4–6 spinal ganglion and anterior horn cells; ↓the expression of neuropeptide Y in spinal ganglion and anterior horn cells	[59]
	Silk sericin	2.4 g/kg/d	5	T2DM rats	↓serum blood glucose concentration; ↓serum growth hormone level; ↓hippocampus growth hormone expression; ↑serum IGF-1 level; ↑IGF-1 and growth hormone receptor in the hippocampus; improved disorders of the growth hormone/IGF-1 axis	[60]
Neuropsychological activity	A syrup made from silk cocoon aqueous extract	5 ml of syrup containing 250 mg dried extract twice daily	12	Patients with mixed anxiety-depressive disorder	↓mean of anxiety and depression scores in week 6 and week 12 compared to control group	[67]
	Silk fibroin nanoparticles	A single dose injection in center of lesion area of the sensori-motor cortex	4 days	Traumatic Brain Injury Model	↓brain damage and recovery of long-term neurological functions; higher viability of primary cell cultures of neurons and astrocytes on silk fibroin matrices under oxygen-glucose deprivation compared to 2D conditions on plastic plates	[61]
	silk fibroin protein enzymatic hydrolysate (FPEH)	0, 280, 400 and 600 mg of FPEH per day in two divided doses	3	Healthy adults with an average age of 55	dose-dependent increases in memory quotient score (MQ), the learning gradient, the numbers of words remembered, the retrieval efficiency, and drawing/recall; The optimal dose for FPEH was 400 or 600 mg, depending on the end point measured; No adverse effects were reported	[62]
	silk amino acid preparation (SAA)	50, 160, or 500 mg/kg	30 days	Parkinson's disease (PD) model rats	Improvement of 6-OHDA-induced impaired pole test performances; improvement of increased using rate of ipsilateral forelimb in cyclinder test and apomorphine-induced circling behavior of PD rats; attenuation of 6-OHDA-induced loss of neurons as well as decreases in dopamine and its metabolites	[65]
Skin health and wound healing activities	Pure silk (100% natural silk) dressing	-	48	Patients with burn wounds covering more than 10% of their total body surface area	↓need for further surgery and scarring compared to patients treated with nylon mesh and collagen; High satisfaction with respect to the aesthetic outcomes; Fast re-epithelization; ↓unpleasant dressing changes frequencies	[69]
	Sodium alginate functionalized with silk sericin/AgNPs hydrogel dressing	Sericin (0.5% w/v); AgNO3 (0.2 mmol/l); Sodium alginate (2% w/v); dressing replacement every 2 days	2	Artificial wound-created in rats	Complete wound healing on day 12; ↓bacterial colony numbers; ↑wound contraction ratio; No pus and no inflammation occurrence	[72]
	AgNPs-sericin/poly (vinyl alcohol) dressing	Sericin (2% w/v); AgNO3 (0.2 mM/l) dressing replacement every 2 days	2	Artificial wound-created in rats	↑wound healing speed; accelerated wound area closure; ↑antibacterial activity	[68]
	SF-based hydrogels	3%/d	8	Rabbit model of hypertrophic scarring	Lighter wound color and closely similar to the surrounding normal skin color in SF hydrogel-treated group; Softened scar texture; ↓scar hyperplasia index	[76]
	silkworm cocoon sol-gel film	-	2	Artificial wound-created in rabbits	↑wounds healing rate; rapid ↓ of the wound size and inflammation; successful reconstruction of intact and thickened epidermis	[78]

AMPKα: AMP-activated protein kinase-α; BrdU: Bromodeoxyuridine; COX-2: Cyclooxygenase 2; Chol: C: Cholesterol; CK-MB: Creatine kinase-MB; DPPH: 2,2-diphenyl-1-picrylhydrazyl; GLK: Germinal center kinase-like kinase; GLUT4: Glucose transporter type 4; G6Pase: Glucose 6-phosphatase; GSH: Glutathione; GSH-PX: Plasma glutathione peroxidase; GSK3β: Glycogen synthase kinase 3 β; HDL: High-density lipoprotein; 4-HNE: 4-hydroxynonenal; HOMA-IR: Homeostatic model assessment; IGF-1: Insulin-like growth factor 1; IL-6: Interleukin 6; ISI: Insulin sensitivity index; LDH: Lactate dehydrogenase; MDA: Malondialdehyde; NF-κB: Nuclear factor kappa light chain enhancer of activated B cells; NP: Nanoparticle; Px: Pancreatectomized; PCK: Phosphoenolpyruvate carboxykinase; PCNA: Proliferating cell nuclear antigen; PFK-1: Phosphofructokinase-1; PI3K: Phosphatidylinositol 3-kinase; PKM-2: Tumor M2-pyruvate kinase; P38MAPK: P38 mitogen-activated protein kinases; PPARγ: Peroxisome proliferator-activated receptor γ; ROS: Reactive oxygen species; SAT: Subcutaneous adipose tissue; SF: Silk fibroin; SOD: Superoxide dismutase; T2DM: Type 2 diabetes mellitus; TG: Triglyceride; TNF-α: Tumor necrosis factor-α; UCP3: Mitochondrial uncoupling protein 3; VAT: Visceral adipose tissue; VLDL: Very-low-density lipoprotein.

### 5.1 Cardioprotective activities

Some studies have looked at the efficacy of silk cocoons on the cardiovascular system. In a preclinical study by Mahmood T et al. [25] on in vivo model, the effect of pretreatment by different formulations of cocoon silk extract, especially Khamira Abresham Sada and Khamira Abresham Hakim Arshad Wala, was studied against Wistar rats induced cardiotoxicity by isoproterenol consumption. The results exhibited the silk formulations were able to suppress heart damage through decreased cardiac marker enzymes (such as CK-MB and troponin), heart weight to body weight ratio, and degree of myonecrosis and filtration of inflammatory cells, as well as improved heart recovery, cardiac antioxidant capacity, and lipid peroxidation. Similarly, the cardioprotective effects of pretreatment by ethanolic extracts of silk cocoon (Abresham) on isoprenaline (ISO)-induced myocardial infarction was shown by decreased inflammation, redness, capillary dilation, and scar formation in histopathologic findings compared to the ISO group. Improved various cardiac enzymes and heart weight/body weight ratio were also reported [26]. In addition, in Wistar albino rats challenged with doxorubicin, a potentially cardiotoxic agent, Khamira Abresham Hakim Arshad Wala pretreatment significantly harnessed cardiac-related enzymatic changes and tissue damages as well as increased antioxidant activities [27].

### 5.2 Antihyperlipidemic and body fat-lowering activities

Sericin has antihyperlipidemic and body fat-lowering effects as it could be used as a potential therapeutic natural protein against obesity. Silk proteins with different fibroin to silk proportions exhibited modified hyperlipidemia, atherogenic index, and the amount of body fat in mice fed with a high-fat diet through increased fecal lipid excretion, lipogenesis inhibition, and adipokine production regulation [28]. In a study by Mir Mahdi A. et al. [29], the effects of *B. Mori* cocoon extract have been examined on the rabbits' model of hyperlipidemia and atherosclerosis. The results demonstrated a marked lipid profile improvement, atherosclerotic plaques size reduction, as well as increased body weight. Another study evaluated the cholesterol-lowering effect of sericin-derived oligopeptides both in vitro and in vivo. This natural product reduced serum total and non-HDL cholesterol along with decreased cholesterol uptake in the monolayer Caco-2 cell line [30]. Likewise, another study with similar results about sericin has been reported [31]. Furthermore, sericin demonstrated its antihyperlipidemic effect by reducing serum triglyceride (TG), total cholesterol, free fatty acids, apolipoproteins rich in cholesterol, liver TG, and the activities of known lipogenic enzymes viz. glucose 6-phosphate dehydrogenase and malic enzyme [32]. In one study, Kunz et al. [33] assessed the anti-obesity potential of sericin in mice-induced obesity by a high-fat diet. Surprisingly, findings indicated that sericin treatment was not fully reverted the biochemical and biometric changes induced by the fat-rich regimen caused obesity; albeit it restored intestinal morphometry and increased lipid excretion in feces at the dose of 1,000 mg/kg.

### 5.3 Antioxidant and antitumor activities

Sericin has long been acknowledged as an antioxidant. It was shown to have a suppressive behavior on lipid peroxidation in the intestine through its antioxidant potential; thereby, it could be exploited to inhibit colon tumor incidence and propagation [14]. In a study using mice model of alcohol-induced liver damage, You-Gui et al. highlighted the capacity of sericin to restore decreased antioxidant enzymes like GSH, GSH-PX, CAT, and SOD to the normal range [34]. Further, sericin has also shown a strong scavenging capacity for hydroxyl, superoxide, and DPPH radicals, as well as antioxidative activity on the peroxidation of linoleic acid [35]. In another in vitro study, different silk sericin hydrolysates revealed significant reducing power and ferrous ion chelating ability compared to the control [36]. Likewise, sericin has demonstrated antioxidant effects in hydrogen peroxide-induced oxidative stress in the feline fibroblast cell line by decreased catalase activity and thiobarbituric acid reactive substances (TBARS). Moreover, Cells treated with sericin exerted marked cell viability and restored cell membrane integrity, highlighting the capacity of sericin to be exploited in cancer therapy [37].

When sericin was fed to the 1,2-dimethylhydrazine-treated mice at 30 g/kg for 115 days, the health beneficiary activities of sericin were revealed in terms of reducing colon adenocarcinoma, cell proliferation, and nitric oxide production [38]. In vitro assay was carried out to investigate the effects of sericin on human colon cancer SW 480 cell line, in two ranges of molecular weight. The small sericin showed higher cell viability decrement effects through increased cell cycle arrest at the S phase and inducing cell apoptosis via the activation of caspase-3 and down-regulation of Bcl-2 expression [39]. Dietary supplementation of the rat model of colon carcinogenesis with 3% sericin considerably lowered the colon's oxidative stress condition and tumor incidence [40]. A study examining the effect of sericin on UV-B-induced acute damage and tumor promotion has reported its capacity to modulate the intensity of red color, area of lesions, and tumor occurrence and metastasis induced by UV-B [41]. Consistently, In two animal model studies, Zhaorigetu S. et al. [42] examined the effect of sericin on tumor promotion in the 7,12-dimethylbenz (alpha) anthracene (DMBA)-initiated and 12-O-tetradecanoylphorbol 13-acetate (TPA)-promoted mouse skin tumorigenesis models. At first, sericin was exploited topically to the skin of DMBA-initiated female ICR mice, followed by tumor progression treatment with TPA. The protective potential of sericin was exhibited through decreased tumor formation and its multiplicity. In the next section, sericin was employed locally to the dorsal mouse skin prior to the double TPA treatment procedure being applied by a 24 h interval. Sericin pre-application markedly suppressed double TPA-induced histological changes indicating inflammatory reactions, comprising cell propagation and leukocyte infiltration. It also alleviated the 4-hydroxynonenal (4-HNE) level and c-fos, c-myc, and COX-2 proteins in normal skin epidermis. Evidence emerging from these studies has implicated the protective potential of silk sericin against skin tumor promotion through bridling oxidative stress and inflammatory responses.

### 5.4 Gastrointestinal protective activity

Valuable research has been performed on the properties of silk cocoons on gastrointestinal activity. An interesting study was conducted by Okazaki Y. et al. [43] in which the effect of sericin was assessed on the intestinal luminal environment in rats fed a high-fat diet. The results showed that sericin had admired effects on reducing colon cancer and ulcerative colitis occurrence via modifying intestinal immune and barrier activities in rats who underwent a high-fat regimen. In another study using mice model of alcohol-induced gastric injury, the administration of sericin was exhibited to restore the disrupted structure of gastric mucosal mitochondria and mucosal abnormalities formed in mouse stomach compared to control via augmentation of antioxidant enzymes activity and increasing "first-pass metabolism" in the liver as well as the enhanced rate of ethanol excretion through the urine [44]. Sericin consumption was also associated with suppressed atropine-induced constipation in rats [45]. Furthermore, oral consumption of sericin significantly increased the gastrointestinal absorption and thus bioavailability of Zn, Fe, Mg, and Ca microelements [46]. In this regard, silk fibroin-based nanoparticles because of their wide biopharmaceutical privileges like biodegradability, biocompatibility, easy self-assembly, low immunogenicity, and simply manageable sequences along with particular functional groups such as imidazole, amino, and carboxyl groups as well as biological activities, namely anti-inflammatory and mucosal healing abilities have been extensively exposed for the treatment of IBD, colon cancer, and reducing chemotherapeutics related side effects [47, 48].

### 5.5 Hypoglycemia and its related complications activities

Blood sugar-lowering effects are among the most popular properties of silk cocoons widely studied. In an in vitro study on insulin-resistant HepG2 cells, silk fibroin peptides increased glucose and lipid metabolisms through augmented glucose consumption and glycogen accumulation along with reduced TG content [49]. In vitro study using a flavonoid-rich ethanolic extract from the green cocoon shell of silkworm exhibited outstanding antioxidant, anti-inflammatory, as well as antihyperglycemic properties, which the latter implemented by inhibition of α-amylase and α-glucosidase, two accused enzymes in increased blood glucose level [8]. In another study, silk peptides demonstrated antidiabetic effects on partial pancreatectomized rats by increased oral glucose tolerance and insulin tolerance as well as insulin secretory activity [50]. Furthermore, dietary silk peptides have a dose-dependent inhibitory effect on blood glucose in high-fat diet-fed mice through the upregulation of GLUT4 and UCP3 pertaining to glucose uptake and mitochondrial function, respectively [51]. After the mice model of type 2 diabetes ate ethanolic extract from the sericin layer for over seven weeks, increased cellular insulin sensitivity and its secretory signaling mediators, improved oral glucose tolerance and insulin tolerance tests, and decreased blood glucose were observed [52]. Moreover, α-glucosidase inhibition [53] increased the expression of key enzymes related to insulin metabolism and glycolysis such as glycogen synthase, GSK3β, GLK, PFK1, PKM2, and AMPKα [54] as well as the capability of pancreatic cell proliferation and regeneration [55] have also been put forth about blood sugar-lowering mechanisms of silk cocoon and its constitutes. Silk cocoon consumption cannot merely bridle the blood sugar levels in diabetic models of hyperglycemia but also has considerable health beneficiary effects on a variety of diabetes-related complications.

In a study exploring the effect of sericin on diabetic nephropathy (DN) in rats, the results depicted that sericin treatment evidently relieved renal pathological changes, decreased the p-p38MAPK inflammatory signaling pathway, which can regulate the inflammatory response of the kidney, and inhibited the activation of NLRP3 inflammasome which possesses a crucial role in DN occurrence and development [56]. Song CJ. et al. [57] studied the effect of sericin on the axis of the testicular growth hormone/insulin-like growth factor-1 in a rat model of type 2 diabetes. As a result, sericin ameliorated spermatogenesis performance and defended the reproductive system against the vulnerable consequences of diabetes. Sericin solution administration indicated a prominent corneal wound healing effect on Otsuka Long-Evans Tokushima fatty rats, a model of diabetic keratopathy [58]. Another study reported that sericin possesses protective potential against diabetes-induced injuries in sciatic-related nerve cells, demonstrated by an increase in neurofilament protein expression in the sciatic nerve and nerve growth factor while the expression of neuropeptide Y in spinal ganglion and anterior horn cells were downregulated [59]. Another study reported that sericin extracted from silk cocoon alleviated hippocampal damage in diabetic rats by its potential in balancing the growth hormone/insulin-like growth factor-1 axis [60].

### 5.6 Neuropsychological activity

Silk fibroin demonstrated neuroprotective activity and restored the neurological status up to 25% after 4 days of injection in a traumatic brain injury model of rats. The growth of primary neuronal cells and astrocytes has been recorded [61]. Oral administration of silk fibroin hydrolysate revealed a significant neuroprotective activity and improved visual and verbal memory in healthy adults [62]. Silk sericin has been reported to be an inhibitor of tyrosinases [63]. In Parkinson's disease, overexpression of tyrosinases causes hydroxylation, which leads to reduction in dopamine content and increase in oxidative stress which both result in neuronal cell death [64]. Silk fibroin peptides preserved the viability of dopaminergic neurons in response to 6-hydroxydopamine neurotoxicity in Parkinson's disease model [65]. Moreover, it has exhibited inhibitory activity against monoamine oxidases (MAOs), important enzymes that cause the breakage of monoamines such as L-DOPA [66]. Administration of a medicinal syrup containing aqueous extract of silkworm cocoon to patients with mixed anxiety-depressive disorder significantly reduced the mean of anxiety and depression scores in weeks 6 and week 12 compared to the control group (P < 0.001) [67]**.**

### 5.7 Skin health and wound healing activities

By virtue of biocompatibility, desired impacts on keratinocytes and fibroblasts, as well as mitogenic and extended moisture retention properties, silk cocoon proteins, particularly sericin, bring forward as a wound-healing agent in dressing materials [68]. A human clinical study investigated the effect of pure silk dressing in treating superficial burn wounds covering more than 10% of the total body surface area. It was cost-effective and provided rapid re-epithelization, prevented unpleasant dressing changes frequencies, and yielded high satisfaction regarding scarring and aesthetic issues [69]. [70, 71] In order to ameliorate sericin antibacterial and physiochemical properties, designing sericin-based composite products using various methods, materials and formulations have been extensively exposed. In this context, a novel semi-interpenetrating hybrid hydrogel containing sodium alginate functionalized with silk sericin and Ag nanoparticles (AgNPs) was fabricated and applied to treat wound injury. The hydrogel showed a phosphate-buffered saline retention ratio of more than 5% after 50 h, a swelling ratio of 32, effective antibacterial activity, and a wound contraction ratio was 99% on day 12 after surgery in the animal experiment [72]. A study by Tao G. et al. [68] was done by developing the in situ biomimetic AgNps embedded silk sericin/polyvinyl alcohol sponges, by which it demonstrated good hygroscopicity, wettability, as well as antibacterial activity and biofilm formation suppression effect towards *Staphylococcus aureus* and *Pseudomonas aeruginosa*. The sponges were expeditiously improved re-epithelization, angiogenesis, and collagen deposition to develop wound healing in the Wistar rats model of skin laceration. In another study, sericin/agarose (50:50) gel-loaded lysozyme synthesized through impregnation method was found to promote wound healing by its distinctive characteristics viz. outstanding water retention capability, superb antimicrobial activity against *Escherichia coli* and *Staphylococcus aureus* with no toxicity on normal cells [73]. Moreover, the wound healing potential of ZnONPs- Polydopamine-coated sericin/PVA composite film formulation was revealed by improved pharmaceutical features like hydrophilicity, swellability, and tensile strength and elongation, along with the antibacterial activity [74]. On the other hand, silk fibroin has held promise for help in skin health compensation and maintenance through increased cell propagation, migration, and adhesion, along with enhanced expression of their related proteins, which are crucial in wound healing [75].

[70]Treatment of burn wound animal model with an injectable hydrogel containing silk fibroin increased wound closure activity, collagen deposition, and angiogenesis [71]. When a silk fibroin-based hydrogel (3%) was exploited in hypertrophic scars, it made them thinner and lighter in color, reduced the density, and caused regular alignment of collagen fibers [76]. In another study by Yu K. et al. [77], silk fibroin sol-gel films indicated accelerated wound healing than standard dressing as well as the successful rebuilding of the epidermis, histologically. Treating with fibroin biomaterials dressing for 10 days decreased the wound size to approximately 30%, further decreased to about 11% after 15 days, whereas these measures in the control group with no treatment were 52% and 49%, respectively [78]. Concerning skin health, a wide variety of new dosage forms for silk and its constituents have been brought into play, including nano-porous films [79], insulin-encapsulated silk fibroin microparticles dressing [80], lyophilized silk fibroin dressing [81], aerosolized nanopowder [82].

## 6 Tissue engineering and drug delivery systems

Thanks to their desirable biocompatibility, tunable mechanical properties, and readily regeneration, silk proteins have been used to formulate various composites for drug delivery and tissue engineering. For this, different techniques, namely, freeze-drying, gas-foaming, salt-leaching, and electrospinning, are exploited to constitute various architectures, like fibers, capsules, spheres, nano-based and porous 3D structures like sponges, hydrogels, foams, and scaffolds which later have been majorly gained momentum in tissue engineering and implantable devices [83, 84]. In the viewpoint of drug delivery, silk fibroin-based formations have been well-established to transport both small (e.g., phytochemicals) and large molecules (e.g., proteins, gene, etc.) reliably, as well as therapeutic agents delivery in a controlled released manner through various pathways (i.e., localized, systemic, or intracellular) in a bare or surface modified forms [84].

Akrami-Hasan-Kohal M. et al. [85] have developed a sustained drug-releasing system of dexamethasone sodium phosphate-loaded chitosan nanoparticles incorporated in silk fibroin hydrogel (SFH/DEX-CSNPs). The results demonstrated a properly interconnected porous matrix structure that sustainedly released the DEX according to the first-order model kinetic over 16 days with a tiny initial burst release. In the subsequent study, an inhalable silk fibroin-based formulation of ciprofloxacin hydrochloride microparticles was fabricated. The employed drug delivery system increased drug loading content, mucosal permeation activity, lung deposition rate, along with outstanding cytocompatibility and biosafety [86]. In addition, efficient delivery of anticancer agents with at least accompanying side effects and safety concerns is the credential of drug carriers in cancer therapy [87]. A recent experiment has addressed this challenge via preparing a pH-responsive nano-based silk sericin decorated zeolitic imidazolate framework-8 to deliver doxorubicin. The formulation endowed superior stability, acid-triggering drug release, efficient cellular internalization, and antitumor activity to doxorubicin [88]. Likewise, another study aimed to evaluate the efficacy of low-molecular-weight polyethyleneimine grafted silk fibroin complex to package plasmid DNA encoded by the inhibitor of growth 4 and interleukin-24 against human lung adenocarcinoma cell line, has been demonstrated not only increased the cell line transfection but also significantly inhibited the cancerous cell proliferation. In addition, no significant toxic effects on normal cells were reported [89].

In recent years, a large body of research has been paid attention to applying silk-based biomaterials to reconstruct organ defects, particularly connective tissues, viz. bone and cartilage [90]. In a study by Lee S. and coworkers, gellan gum/silk fibroin/chondroitin sulfate (GG/SF/CS) ternary injectable hydrogels with different proportions of the component to cartilage tissue regenerating were fabricated. The hydrogels ' striking mechanical and physicochemical features were confirmed regarding findings obtained from the swelling test, degradation rate, compression test, and proper shear thinning property. The 0.5 GG/3.5% SF/CS formulation was chosen for in vitro and in vivo studies. The encapsulated chondrocytes in the hydrogel showed low toxicity, improved cell adhesion and proliferation, and expression of cartilage-specific extracellular matrix (ECM) and genes (SOX9, COL-2, and AGG) [91]. Recent in vitro study evaluating the endochondral ossification of decellularized cartilage-derived extracellular matrix (CD-ECM) incorporated silk fibroin scaffold containing human bone marrow-derived mesenchymal stem cells (hBMSC's) have reported its ability to markedly impact on early and late hypertrophic phases of the cells differentiation as well as increased expression of osteogenic markers, including IBSP, OSX, and COL1A1 by 2, 15, and 3 folds compared to the scaffold-free of CD-ECM. Likewise, increased calcium deposition, alkaline phosphatase secretion, and retention of sulfated glycosaminoglycan content as a consequence of using the CD-ECM/silk fibroin scaffold were revealed [92].

## 7 Potential toxicity and possible side effects

Silk cocoon, majorly composed of biocompatible ingredients, has been widely exploited since ancient times; however, there is a paucity of recorded data regarding its toxicological aspects. In addition to US FDA approval of silk suture, noteworthy, fibroin engineered biological scaffold called SERI® has been exploited for soft tissue support, especially abdominal wall repair [93, 94].

The safety of water-extract sericin has been studied using different preclinical approaches comprising genotoxicity assays (the bacterial reverse mutation test, the mammalian erythrocyte micronucleus test, and chromosomal aberration test of the mouse spermatogonia), as well as subchronic toxicity evaluation of Sprague-Dawley rats, during 90 days. According to the results obtained from the abovementioned genotoxicity assays, sericin exhibited nonmutagenic and nongenotoxic effects compared to the negative control both in vitro and in vivo. During the 90-day subchronic exposure, no notable treatment-associated deaths were recorded. The changes in the body and organ weight, food consumption, blood hematology and biochemistry factors, urine indexes, and histopathological findings were not significant. The no-observed-adverse-effect level (NOAEL) measure of the studied sericin was determined to be 1 g/kg/day in both sex rats [95]. Further, the same tests were employed to study the safety profile of silkworm extract powder in rats; thereby, the lethal dose once oral administration was more than 5000 mg/kg, and no adverse effects were reported during the 90-day consumption of the powder. Ultimately, the NOAEL of silkworm powder extract was estimated to be 2000 mg/kg/day in both male and female rats [96]. The results obtained from the MTT assay exhibited that silk sericin solutions from different extraction methods showed no toxic effects towards mouse fibroblast cells at concentrations up to 40 μg/mL after 24 h, although the principle was violated in some extraction methods at higher concentrations [97]. Another study evaluating the silk protein film safety exhibited no abnormal clinical signs, changes in biochemical and gross pathological parameters, erythema, edema, scar, and skin reactions compared with the control group [98]. Besides, the safety profile of silk and fibroin in concentrations used in cosmetics has been exhaustively studied [99]. Acute toxicity induced by sericin consumption once orally at doses of 500, 1000, and 2000 mg/kg did not significantly alter any of the measured biological parameters such as difference in weight, the quality of excreted feces, rate of respiratory and visual characteristics, during 14 days. On the other hand, in sub-acute toxicity evaluation with the same doses for four weeks, no noticeable toxicological effects were detected, though some inflammation or abnormalities in the crucial organs just at the max dose were reported. Eventually, it was estimated that the NOAEL is below 2000 mg/kg, which can be accounted safe [100]. Nevertheless, there are some reports regarding adverse effects arising from silk and its ingredients. There have been reports of the increased inflammatory response following silk consumption, which could be lessened by using the rigorous procedure to isolate pure fibroin [93]. Further, silk fibroin occasionally promotes amyloidogenesis, howbeit possesses a lesser potential for amyloidosis [101]. In addition, in a cross-sectional study on children living in a rural sericulture province of China, silk sensitization related to childhood asthma pathogenesis and severity was firstly reported [102].

## 8 Conclusion

Silk cocoon is one of the well-known naturally occurring agents with several therapeutic activities. These effects are sometimes similar to those proposed in ethnomedicine. One of the most important effects of silk cocoon in traditional medicine is the strong emphasis on the heart's effect and helps maintain cardiovascular health. New studies have also confirmed these effects and demonstrated the cardioprotective activity of silk. In traditional medicine, silk cocoon is also known as a tonic for the liver and relieves hepatic obstruction and liver weakness. Current studies have also confirmed its protective effect on the liver. Silk has been introduced in traditional medicine as a tonic for general body weakness, which, if proven scientifically, maybe due to the protein matrix and the variety of amino acids that make up sericin.

In the gastrointestinal system, the silk cocoon has mentioned strengthening and improving digestion. Recent studies have shown that silk can enhance gastric mucosa, but no studies have been done on the effect of silk on digestion. The silk has been claimed to heal the wound inside the eye, enhance the eye's vision, and effectively maintain the eye's health. The effect of corneal wound healing has been confirmed by silk, which is due to the sericin protein.

Although various studies have been performed on silkworm cocoon and its constituents, sericin and fibroin, further in vivo and clinical trials are required to evaluate the exact underlying mechanisms of action as well as their pharmacological properties.

The therapeutic effect of silk in diabetes and polydipsia has been mentioned in traditional medicine, and new research has confirmed silk's protective effect in diabetes.

Traditional medicine has reported many neuropsychological effects from silk, including antidepressant, anxiolytic, and memory-enhancing effects. Various studies have demonstrated the effects of silk on neurodegenerative disorders including Alzheimer's disease and Parkinson's disease. The silk fibroin peptides improved acetylcholine concentration and cognitive response and enhanced memorial activity. Moreover, the neuroprotective activity of silk sericin has been shown in Alzheimer's disease. The amino acid sequence and the by-products generated after silk degradation exhibited antioxidant and anti-inflammatory properties as well as affected different pathways involved in the pathogenesis of neurodegenerative disorders. Moreover, biomaterial properties of silk by itself (scaffold structure and biomechanical features) give it the capacity to interact with the target tissue providing tissue and cellular anchorage, stem cells, and/or drug delivery [103]. The theory in traditional medicine is that the heart produces the animal spirit, and the animal spirit enhances the physical spirit, and the natural spirit, and the spiritual spirit means the brain, and the natural spirit means the liver, and so the drugs that enhance the animal spirit That is, the heart is followed by the strengthening of the physical and natural spirits, which strengthen the spiritual soul to bring about happiness and relieve depression, which is why people with heart problems also show symptoms of depression and so Silk can be used in neurological studies.

The anticancer effect mentioned for silk associated with colon cancer may be related to the antioxidant effect that silk has shown itself. In traditional medicine, it has also been mentioned that silk effectively removes soda from the digestive tract. In traditional medicine, soda is one of the leading causes of cancer, so the anticancer effect of the new articles partly confirms the effects mentioned in traditional medicine.

Historically, silkworm silk has been used by the textile industry for thousands of years due to its excellent physical properties, such as lightweight, high mechanical strength, flexibility, and luster. Recently, silk has been considered as a sustainable, biodegradable material platform that can be used in a wide variety of biomedical applications, such as medicine, biotechnology and filtration. The mechanical robustness, transparency and surface flatness of silk films are compelling features for photonics and electronics applications. With advances in nanotechnology, new and innovative silk-based material formats are being developed, including nanoparticles, nanofibers, nano-pattered films, hydrogels and aligned scaffolds. The direct utilization and re-engineering of silk-based biomaterials into medical and technological material platforms is already underway in many laboratories and offers a path forward for “green bio-nanotechnology”. No other synthetically or biologically derived polymer systems are available that possess this range of extraordinary material properties and biological interfaces. Therefore, silk remains as a model biopolymer to study and also a prime candidate for bio-devices. Further, the FDA has approved silk medical devices for sutures and as a support structure during reconstructive surgery [104].

Since for nanomedicine applications, most studies have been focused on the development of new technologies associated with biocompatibility assays, Further studies devoted to intracellular mechanisms of action is highly recommended. These types of studies help us to enhance therapeutic activities of nanoformulations and to promote synergistic effects with chemotherapeutic agents. Moreover, studies on immunogenicity, pharmacokinetics, and toxicological aspects will provide precise information for designing clinical trials.

## Declarations

### Author contribution statement

All authors listed have significantly contributed to the development and the writing of this article.

### Funding statement

This study has been partially supported by Tehran University of Medical Sciences; Grant No. 98-03-147-45414.

### Data availability statement

No data was used for the research described in the article.

### Declaration of interest's statement

The authors declare no conflict of interest.

### Additional information

No additional information is available for this paper.

## References

[1] Kundu B. (2013). Silk fibroin biomaterials for tissue regenerations. Adv. Drug Deliv. Rev..

[2] Kunz R.I. (2016). Silkworm sericin: properties and biomedical applications. BioMed Res. Int..

[3] Ahsan F. (2018). An insight on silk protein sericin: from processing to biomedical application. Drug Res..

[4] Aramwit P., Siritientong T., Srichana T. (2012). Potential applications of silk sericin, a natural protein from textile industry by-products. Waste Manag. Res..

[5] Zhang Y.Q. (2002). Applications of natural silk protein sericin in biomaterials. Biotechnol. Adv..

[6] Wang W.H. (2021). Functionality of silk cocoon (Bombyx mori L.) sericin extracts obtained through high-temperature hydrothermal method. Materials.

[7] Das G. (2021). Sericin based nanoformulations: a comprehensive review on molecular mechanisms of interaction with organisms to biological applications. J. Nanobiotechnol..

[8] Wang H.Y., Zhao J.G., Zhang Y.Q. (2020). The flavonoid-rich ethanolic extract from the green cocoon shell of silkworm has excellent antioxidation, glucosidase inhibition, and cell protective effects in vitro. Food Nutr. Res..

[9] Chouhan D., Mandal B.B. (2020). Silk biomaterials in wound healing and skin regeneration therapeutics: from bench to bedside. Acta Biomater..

[10] Prasong S., Yaowalak S., Wilaiwan S. (2009). Characteristics of silk fiber with and without sericin component: a comparison between Bombyx mori and Philosamia ricini silks. Pakistan J. Biol. Sci..

[11] Siritientong T. (2016). The effects of Bombyx mori silk strain and extraction time on the molecular and biological characteristics of sericin. Biosci. Biotechnol. Biochem..

[12] Mondal M., Trivedy K., Irmal Kumar S. (2007). The silk proteins, sericin and fibroin in silkworm, Bombyx mori Linn., A review %J. Caspian J. Environ. Sci..

[13] Rajput S.K. (2015).

[14] Cao T.T., Zhang Y.Q. (2016). Processing and characterization of silk sericin from Bombyx mori and its application in biomaterials and biomedicines. Mater. Sci. Eng. Mater. Biol. Appl..

[15] Huang Y. (2016). Reference Module in Food Science.

[16] Zhao J.G., Zhang Y.Q. (2016). A new estimation of the total flavonoids in silkworm cocoon sericin layer through aglycone determination by hydrolysis-assisted extraction and HPLC-DAD analysis. Food Nutr. Res..

[17] Zhou G.X. (2007). [Alkaloid constituents from silkworm dropping of Bombyx mori]. Zhong Yao Cai.

[18] Khan M.S. (2014). Scientific validation of cardioprotective attribute by standardized extract of Bombyx mori against doxorubicin-induced cardiotoxicity in murine model. Excli. J..

[19] Kaskoos R., Ali M., Naquvi K. (2012).

[20] Hirayama C. (2006). C-prolinylquercetins from the yellow cocoon shell of the silkworm, Bombyx mori. Phytochemistry.

[21] Good I.L., Kenoyer J.M., Meadow R.H. (2009).

[22] Parveen A., Nigar Z. (2017). AABRESHAM (BOMBYX mori): a BOON to medical science for the prevention of atherosclerosis. Indian J. Pharmaceut. Sci..

[23] Dixit A. (2010). Ethno-medico-biological studies of South India. Indian J. Tradit. Knowl..

[24] Wang X. (2020). Novel mulberry silkworm cocoon-derived carbon dots and their anti-inflammatory properties. Artif. Cell Nanomed. Biotechnol..

[25] Mahmood T. (2015).

[26] Srivastav R.K. (2013). Evaluation of cardioprotective effect of silk cocoon (Abresham) on isoprenaline-induced myocardial infarction in rats. Avicenna J. Phytomed..

[27] Nazmi A.S. (2011). Protective effects of 'Khamira Abresham Hakim Arshad Wala', a unani formulation against doxorubicin-induced cardiotoxicity and nephrotoxicity. Toxicol. Mech. Methods.

[28] Seo C.W. (2011). Antihyperlipidemic and body fat-lowering effects of silk proteins with different fibroin/sericin compositions in mice fed with high fat diet. J. Agric. Food Chem..

[29] Ali M.M., Arumugam S.B. (2011). Effect of crude extract of Bombyx mori coccoons in hyperlipidemia and atherosclerosis. J. Ayurveda Integr. Med..

[30] Lapphanichayakool P., Sutheerawattananonda M., Limpeanchob N. (2017). Hypocholesterolemic effect of sericin-derived oligopeptides in high-cholesterol fed rats. J. Nat. Med..

[31] Limpeanchob N. (2010). Sericin reduces serum cholesterol in rats and cholesterol uptake into Caco-2 cells. J. Agric. Food Chem..

[32] Okazaki Y. (2010). Consumption of sericin reduces serum lipids, ameliorates glucose tolerance and elevates serum adiponectin in rats fed a high-fat diet. Biosci. Biotechnol. Biochem..

[33] Kunz R.I. (2020). Sericin as treatment of obesity: morphophysiological effects in obese mice fed with high-fat diet. Einstein (Sao Paulo).

[34] Li Y.G. (2008). Protective effects of sericin protein on alcohol-mediated liver damage in mice. Alcohol Alcohol.

[35] Fan J.-b. (2009).

[36] Fan J.-B. (2010).

[37] Jagtapb S.G., Khyade V.B. (2016).

[38] Zhaorigetu S. (2001).

[39] Kaewkorn W. (2012). Effects of silk sericin on the proliferation and apoptosis of colon cancer cells. Biol. Res..

[40] Zhaorigetu S., Sasaki M., Kato N. (2007). Consumption of sericin suppresses colon oxidative stress and aberrant crypt foci in 1,2-dimethylhydrazine-treated rats by colon undigested sericin. J. Nutr. Sci. Vitaminol..

[41] Zhaorigetu S. (2003). Inhibitory effects of silk protein, sericin on UVB-induced acute damage and tumor promotion by reducing oxidative stress in the skin of hairless mouse. J. Photochem. Photobiol., B.

[42] Zhaorigetu S. (2003). Silk protein, sericin, suppresses DMBA-TPA-induced mouse skin tumorigenesis by reducing oxidative stress, inflammatory responses and endogenous tumor promoter TNF-alpha. Oncol. Rep..

[43] Okazaki Y. (2011). Consumption of a resistant protein, sericin, elevates fecal immunoglobulin A, mucins, and cecal organic acids in rats fed a high-fat diet. J. Nutr..

[44] Li Y.G. (2008). Protective effect of sericin peptide against alcohol-induced gastric injury in mice. Chin. Med. J..

[45] Sasaki M. (2000).

[46] Sasaki M., Yamada H., Kato N.J.N.R. (2000).

[47] Hudita A. (2021). Bioinspired silk fibroin nano-delivery systems protect against 5-FU induced gastrointestinal mucositis in a mouse model and display antitumor effects on HT-29 colorectal cancer cells in vitro. Nanotoxicology.

[48] Khosropanah M.H. (2021). Biomedical applications of silkworm (Bombyx Mori) proteins in regenerative medicine (a narrative review). J. Tissue Eng. Regen. Med..

[49] Yang Q. (2019). The protective effect of silk fibroin on high glucose induced insulin resistance in HepG2 cells. Environ. Toxicol. Pharmacol..

[50] Park S. (2020). Acid hydrolyzed silk peptide consumption improves anti-diabetic symptoms by potentiating insulin secretion and preventing gut microbiome dysbiosis in non-obese type 2 diabetic animals. Nutrients.

[51] Lee K. (2020). Effect of dietary silk peptide on obesity, hyperglycemia, and skeletal muscle regeneration in high-fat diet-fed mice. Cells.

[52] Zhao J.G. (2019). Therapeutic effects of ethanolic extract from the green cocoon shell of silkworm Bombyx mori on type 2 diabetic mice and its hypoglycaemic mechanism. Toxicol. Res..

[53] Fang Y. (2017). The kinetics and mechanism of α-glucosidase inhibition by F5-SP, a novel compound derived from sericin peptides. Food Funct..

[54] Dong X. (2020). Silk sericin has significantly hypoglycaemic effect in type 2 diabetic mice via anti-oxidation and anti-inflammation. Int. J. Biol. Macromol..

[55] Park S.Y. (2020). Silk fibroin promotes the regeneration of pancreatic β-cells in the C57BL/KsJ-Lepr(db/db) mouse. Molecules.

[56] Liu D. (2020). Effect of sericin on the p38MAPK signaling pathway and NLRP3 inflammasome in the kidney of type 2 diabetic rats. Exp. Ther. Med..

[57] Song C.J. (2015). Effects of sericin on the testicular growth hormone/insulin-like growth factor-1 axis in a rat model of type 2 diabetes. Int. J. Clin. Exp. Med..

[58] Nagai N., Ito Y. (2013). Therapeutic effects of sericin on diabetic keratopathy in Otsuka Long-Evans Tokushima Fatty rats. World J. Diabetes.

[59] Song C. (2013). Sericin protects against diabetes-induced injuries in sciatic nerve and related nerve cells. Neural. Regen. Res..

[60] Chen Z. (2013). Effect of sericin on diabetic hippocampal growth hormone/insulin-like growth factor 1 axis. Neural. Regen. Res..

[61] Moisenovich M.M. (2019). Effect of silk fibroin on neuroregeneration after traumatic brain injury. Neurochem. Res..

[62] Kang Y.K. (2018). Effect of a fibroin enzymatic hydrolysate on memory improvement: a placebo-controlled, double-blind study. Nutrients.

[63] Aramwit P. (2010). Properties and antityrosinase activity of sericin from various extraction methods. Biotechnol. Appl. Biochem..

[64] Shahpiri Z. (2016). Phytochemicals as future drugs for Parkinson's disease: a comprehensive review. Rev. Neurosci..

[65] Kim T. (2011). Tyrosine-fortified silk amino acids improve physical function of Parkinson's disease rats. Food Sci. Biotechnol..

[66] Banagozar Mohammadi A. (2019). Identification and applications of neuroactive silk proteins: a narrative review. J. Appl. Biomed..

[67] Zeinalpour Y. (2021). Effect of medicinal syrup made from silkworm cocoon on mixed anxiety-depression disorder: a triple-blind randomized clinical trial. Iran. Red Crescent Med. J..

[68] Tao G. (2019). Bioinspired design of AgNPs embedded silk sericin-based sponges for efficiently combating bacteria and promoting wound healing. Mater. Des..

[69] Schiefer J.L. (2020). Feasibility of pure silk for the treatment of large superficial burn wounds covering over 10% of the total body surface. J. Burn Care Res..

[70] Lee W.Y. (2014). Effectiveness of woven silk dressing materials on full-skin thickness burn wounds in rat model. Maxillofac. Plast. Reconstr. Surg..

[71] Dong M. (2021). Novel fabrication of antibiotic containing multifunctional silk fibroin injectable hydrogel dressing to enhance bactericidal action and wound healing efficiency on burn wound: in vitro and in vivo evaluations. Int. Wound J..

[72] Tao G. (2021). Fabrication of antibacterial sericin based hydrogel as an injectable and mouldable wound dressing. Mater. Sci. Eng. Mater. Biol. Appl..

[73] Yang M. (2018). Fabrication of sericin/agrose gel loaded lysozyme and its potential in wound dressing application. Nanomaterials.

[74] Ai L. (2019). Polydopamine-based surface modification of ZnO nanoparticles on sericin/polyvinyl alcohol composite film for antibacterial application. Molecules.

[75] Vidya M., Rajagopal S. (2021). Silk fibroin: a promising tool for wound healing and skin regeneration. Int. J. Poly. Sci..

[76] Li Z. (2020). Topical application of silk fibroin-based hydrogel in preventing hypertrophic scars. Colloids Surf. B Biointerfaces.

[77] Guan Y. (2020). Silk fibroin hydrogel promote burn wound healing through regulating TLN1 expression and affecting cell adhesion and migration. J. Mater. Sci. Mater. Med..

[78] Yu K. (2017). Accelerated wound-healing capabilities of a dressing fabricated from silkworm cocoon. Int. J. Biol. Macromol..

[79] Liu J. (2021). Generation of nano-pores in silk fibroin films using silk nanoparticles for full-thickness wound healing. Biomacromolecules.

[80] Li X. (2017). Functionalized silk fibroin dressing with topical bioactive insulin release for accelerated chronic wound healing. Mater. Sci. Eng. Mater. Biol. Appl..

[81] Chou K.C. (2021). Cutaneous regeneration mechanism of β-sheet silk fibroin in a rat burn wound healing model. Polymers.

[82] Karaly A.H., Sarhan W.A., El-Sherbiny I.M. (2021). Development of a silk fibroin-based multitask aerosolized nanopowder formula for efficient wound healing. Int. J. Biol. Macromol..

[83] Cheng G. (2018). Advanced silk fibroin biomaterials for cartilage regeneration. ACS Biomater. Sci. Eng..

[84] Wani S.U.D. (2020). Silk fibroin as a natural polymeric based bio-material for tissue engineering and drug delivery systems-A review. Int. J. Biol. Macromol..

[85] Akrami-Hasan-Kohal M., Eskandari M., Solouk A. (2021). Silk fibroin hydrogel/dexamethasone sodium phosphate loaded chitosan nanoparticles as a potential drug delivery system. Colloids Surf. B Biointerfaces.

[86] Lin L. (2021). The rough inhalable ciprofloxacin hydrochloride microparticles based on silk fibroin for non-cystic fibrosis bronchiectasis therapy with good biocompatibility. Int. J. Pharm..

[87] Nguyen T.P. (2019). Silk fibroin-based biomaterials for biomedical applications: a review. Polymers.

[88] Li X. (2021). Engineering silk sericin decorated zeolitic imidazolate framework-8 nanoplatform to enhance chemotherapy. Colloids Surf. B Biointerfaces.

[89] Niu L. (2021). Polyethylenimine-modified Bombyx mori silk fibroin as a delivery carrier of the ING4-IL-24 coexpression plasmid. Polymers.

[90] Zuluaga-Vélez A. (2021). Silk fibroin nanocomposites as tissue engineering scaffolds - a systematic review. Biomed. Pharmacother..

[91] Lee S. (2021). Development and evaluation of gellan gum/silk fibroin/chondroitin sulfate ternary injectable hydrogel for cartilage tissue engineering. Biomolecules.

[92] Jeyakumar V. (2021). Decellularized cartilage extracellular matrix incorporated silk fibroin hybrid scaffolds for endochondral ossification mediated bone regeneration. Int. J. Mol. Sci..

[93] Madden P.W., Klyubin I., Ahearne M.J. (2020). Silk fibroin safety in the eye: a review that highlights a concern. BMJ Open Ophthalmol..

[94] Jewell M. (2015). The development of SERI® Surgical Scaffold, an engineered biological scaffold. Ann. N. Y. Acad. Sci..

[95] Qin H. (2020). Safety assessment of water-extract sericin from silkworm (Bombyx mori) cocoons using different model approaches. BioMed Res. Int..

[96] Heo H.S. (2013). Evaluation of general toxicity and genotoxicity of the silkworm extract powder. Toxicol. Res..

[97] Aramwit P. (2010). The effect of sericin from various extraction methods on cell viability and collagen production. Int. J. Mol. Sci..

[98] Padol A.R. (2011). Safety evaluation of silk protein film (a novel wound healing agent) in terms of acute dermal toxicity, acute dermal irritation and skin sensitization. Toxicol. Int..

[99] Johnson W. (2020). Safety assessment of silk protein ingredients as used in cosmetics. Int. J. Toxicol..

[100] Ahsan F. (2020). Diligent profiling of preclinical safety of the silk protein sericin. J. Basic Clin. Physiol. Pharmacol..

[101] Tsukawaki S. (2016). Studies on the potential risk of amyloidosis from exposure to silk fibroin. Biomed. Mater..

[102] Celedón J.C. (2001). Sensitization to silk and childhood asthma in rural China. Pediatrics.

[103] Yonesi M. (2021). Silk fibroin: an ancient material for repairing the injured nervous system. Pharmaceutics.

[104] Huang W. (2018). Silkworm silk-based materials and devices generated using bio-nanotechnology. Chem. Soc. Rev..

